# Phytonutrients, Colorant Pigments, Phytochemicals, and Antioxidant Potential of Orphan Leafy *Amaranthus* Species

**DOI:** 10.3390/molecules27092899

**Published:** 2022-05-02

**Authors:** Umakanta Sarker, Md. Golam Rabbani, Shinya Oba, Wagdy M. Eldehna, Sara T. Al-Rashood, Nada M. Mostafa, Omayma A. Eldahshan

**Affiliations:** 1 Department of Genetics and Plant Breeding, Faculty of Agriculture, Bangabandhu Sheikh Mujibur Rahman Agricultural University, Gazipur 1706, Bangladesh; 2 Department of Horticulture, Faculty of Agriculture, Bangladesh Agricultural University, Mymensingh 2202, Bangladesh; drmgrabbani@yahoo.com; 3 Laboratory of Field Science, Faculty of Applied Biological Sciences, Gifu University, Yanagido 1-1, Gifu 501-1193, Japan; soba@gifu-u.ac.jp; 4 School of Biotechnology, Badr University in Cairo, Badr City 11829, Egypt; wagdy2000@gmail.com; 5 Department of Pharmaceutical Chemistry, Faculty of Pharmacy, Kafrelsheikh University, Kafrelsheikh 33516, Egypt; 6 Department of Pharmaceutical Chemistry, College of Pharmacy, King Saud University, P.O. Box 2457, Riyadh 11451, Saudi Arabia; salrashood@ksu.edu.sa; 7 Department of Pharmacognosy, Faculty of Pharmacy, Ain Shams University, Cairo 11566, Egypt; nadamostafa@pharma.asu.edu.eg (N.M.M.); oeldahshan@pharma.asu.edu.eg (O.A.E.)

**Keywords:** underutilized leafy vegetables, proximate composition, minerals, antioxidant pigments, polyphenols, flavonoids, vitamin C, DPPH, antioxidant activity, ABTS^+^

## Abstract

The underutilized *Amaranthus* leafy vegetables are a unique basis of pigments such as β-cyanins, β-xanthins, and betalains with radical scavenging capacity (RSC). They have abundant phytonutrients and antioxidant components, such as pigments, vitamins, phenolics, and flavonoids. Eight selected genotypes (four genotypes from each species) of underutilized *Amaranthus* leafy vegetables were evaluated for phytonutrients, pigments, vitamins, phenolics, flavonoids, and antioxidants in a randomized complete block design under ambient field conditions with three replicates. The studied traits showed a wide range of variations across eight genotypes of two species of *Amaranthus* leafy vegetables. The highest fat, β-xanthins, K, dietary fiber, Mg, β-cyanins, Mn, chlorophyll *ab*, Zn, TP, TF, betalains, chlorophyll *a* content, and (RSC) (DPPH) and RSC (ABTS^+^) were obtained from *A. tricolor* accessions. Conversely, the highest protein, Cu, carbohydrates, Ca, and chlorophyll *b* content were obtained from *A. lividus* accessions. The highest dry matter, carotenoids, Fe, energy, and ash were obtained from *A. tricolor* and *A. lividus*. The accession AT2 confirmed the highest vit. C and RSC (DPPH) and RSC (ABTS+); AT5 had the highest TP content; and AT12 had the highest TF content. *A. tricolor* accessions had high phytochemicals across the two species, such as phytopigments, vitamins, phenolics, antioxidants, and flavonoids, with considerable nutrients and protein. Hence, *A. tricolor* accessions can be used as high-yielding cultivars comprising ample antioxidants. The correlation study revealed that vitamin C, pigments, flavonoids, β-carotene, and phenolics demonstrated a strong RSC, and showed a substantial contribution to the antioxidant potential (AP) of *A. tricolor*. The investigation exposed that the accessions displayed a plentiful origin of nutritional values, phytochemicals, and AP with good quenching ability of reactive oxygen species (ROS) that provide enormous prospects for nourishing the mineral-, antioxidant-, and vitamin-threatened community.

## 1. Introduction

Amaranth is a promising crop with widespread divergence [1,2,3,4,5,6,7]. Across seventy species of the family of Amaranthaceae, 17 are consumed as vegetables, and 3 are consumed as grains [8]. Amaranth is a fast-growing C_4_ plant with versatile uses, including vegetables, ornamentals, and grains. It is a widely acclimated vegetable with a widespread distribution in America, Australia, Africa, Europe, and Asia. It is a low-cost vegetable whose edible stems and leaves have abundant protein, including methionine and lysine (important amino acids for humans) [9,10,11,12]; digestible fiber; ascorbic acid; carotenoids; and minerals containing Ca, Cu, Mg, Zn, K, Fe, and Mn [13,14,15,16,17,18,19,20,21,22,23,24,25,26,27,28,29,30,31,32,33,34,35,36,37]. *Amaranthus* leafy vegetables are used as many traditional medicines, especially antimicrobial [38,39,40,41,42,43,44,45,46,47,48], anthelmintic [49,50,51,52], antiviral [53], neuroprotective [54], anti-inflammatory [55,56], antiulcer [57], anticancer [58,59,60], hepatoprotective [61,62,63,64], anti-hyperlipidemic [65,66,67,68,69,70], antidiabetic, antidepressant, antimalarial activities, and snake antidotes [71,72,73,74,75,76]. It also has abundant phytopigments, including β-cyanins, anthocyanin, β-xanthins, betalains, carotenoids, and chlorophylls [77,78,79,80,81,82,83,84] with high RSC [85,86,87,88,89,90,91,92,93,94,95]. It also has sufficient phytochemicals, including ascorbic acids, phenolic acids, and flavonoids [96,97,98,99,100,101,102] and AP [103,104,105,106,107,108,109,110,111,112,113,114,115,116,117,118,119,120]. These compounds of natural origin quench ROS [121,122,123,124,125,126,127,128,129,130,131,132,133,134,135,136,137,138,139], and predominantly influence the industry of foods [140,141]. Phytopigments, including β-cyanins, carotenoids, β-xanthins, betanin, and amaranthine, have the important RSC [141]. They are broadly acclimated to different abiotic stresses such as water deficits [142,143,144,145,146,147,148,149,150] and salinization [151,152,153,154].

There is significant variability and phenotypic plasticity of *Amaranthus* germplasms in Asia, Africa, Bangladesh, and South America [155], with versatile usages. In Africa and South-East Asia, including India and Bangladesh, amaranth is a cheap and popular leafy vegetable. The flavor, taste, and beautiful color make it a typical leafy vegetable in Asia and many global regions. It grows year-round in Bangladesh, including the leafy vegetables gap in summer [19,20].

Nowadays, both consumers and researchers give attention to consuming vegetables for achieving antioxidants of natural origin [156,157,158,159]. *Amaranthus* has significant groups of natural antioxidants, such as β-cyanin, β-xanthin, amaranthine, betalain, phenolics, carotenoids, vitamin C, and flavonoids [140,141]. These natural antioxidant phytochemicals defend against many diseases, such as atherosclerosis, emphysema, cardiovascular diseases, cancer, cataracts, arthritis, retinopathy, and degenerative diseases of neurons, and contribute significantly to human health promotion [160,161,162]. Natural products with AP have an extensive interest. Numerous medicinal plants having interesting AP have been proposed as protective capacities because of constituents’ presence, including vitamins C, flavonoids, carotenoids, phenolics, and other non-nutrient components [163].

In the current years, our research group is investigating the opportunity of applying leafy vegetable amaranth as a foundation of natural pigments because of their ample β-cyanins, β-xanthins, betalains color pigments, and phytochemicals of interest in the food industry [77,78]. Our research group carefully chose a few high-yielding AP *A. tricolor* and *A. lividus* cultivars from the gene pool in our earlier studies [19,20,77,78]. This study evaluates the comparative performance of the selected eight best genotypes of two species of *A. tricolor* and *A. lividus*, in terms of minerals, nutrients, antioxidants phytochemicals, and pigments having high AP.

## 2. Materials and Methods

### 2.1. Materials

The seeds of eight accessions of *A. lividus* and *A. tricolor* were selected from our earlier collected germplasms.

### 2.2. Layout and Design

The study was implemented at Bangabandhu Sheikh Mujibur Rahman Agricultural University using a completely randomized block design (RCBD) in three replicates. Each experimental plot comprises a 1 m^2^ area with 20 cm rows and a 5 cm plant distance.

### 2.3. Management Practices

We applied 150 kg MP, 200 kg urea, 100 kg TSP, 60 kg gypsum, and 10 compost (cowdung:straw = 1:2 *w*/*w*) per ha of land. We maintained suitable cultural management. Thinning was performed to keep the particular space of plants in a row. The experimental plots were kept free from weeds by applying appropriate and regular weeding and hoeing. We applied regular irrigation in the plot for appropriate growth of the crop. Leaf samples were collected from 30-day-old plants.

### 2.4. Solvent and Reagents

Solvent: Methanol and acetone. Reagents: cesium chloride, dithiothreitol (DTT), HClO_4_, ascorbic acid, HNO_3_, H_2_SO_4_, and Trolox, ABTS^+^, Folin–Ciocalteu reagent, DPPH, gallic acid, 2, 2-dipyridyl, rutin, hexahydrate aluminum chloride, K acetate, sodium carbonate, and K persulfate.

### 2.5. Determination of Proximate Composition

Crude fat, ash, moisture, crude protein, fiber, and energy (gross) were estimated according to the Association of Official Agricultural Chemists (AOAC) method [164]. We followed the Micro-Kjeldahl method to calculate the nitrogen, and finally, crude protein was measured by multiplying nitrogen by 6.25 (AOAC method 976.05). The crude protein, total moisture, ash, and crude fat (%) were subtracted from 100 for calculating carbohydrate (g 100 g^−1^ FW).

### 2.6. Determination of Minerals

In an oven, the leaves were dried out for 24 h at 70 °C. Dried samples were ground in a mill. We determined Ca, K, Mg, Fe, Mn, Cu, and Zn using the nitric and perchloric acid method [165]. A dried leaf sample (0.5 g) was digested with 10 mL H_2_SO_4_ (96%), 400 mL HNO_3_ (65%), and 40 mL HClO_4_ (70%) in the presence of carborundum beads. A blue-colored phosphomolybdenum complex was formed by adding antimony and ascorbic acid to the complex solution (yellow). Atomic absorption spectrophotometry (AAS) (Hitachi, Tokyo, Japan) was used to read the absorbance at 213.9 nm (Zn), 285.2 nm (Mg), 279.5 nm (Mn), 76 6.5 nm (K), 422.7 nm (Ca), 248.3 nm (Fe), and 324.8 nm (Cu).

### 2.7. Estimation of Carotenoids and Chlorophylls

The leaf samples were added in acetone (80%) to determine carotenoids, and chlorophyll *a*, *ab*, and *b* [165]. Carotenoids, chlorophyll *b*, and chlorophyll *a* were calculated by measuring the absorbance at 470, 663, and 646 nm, respectively, using a spectrophotometer (Hitachi, Japan).

### 2.8. β-Cyanins and β-Xanthins Content Measurement

β-Cyanins and β-xanthins were measured by extracting the leaf samples in 80% MeOH containing 50 mM ascorbic acid [166,167]. β-cyanins and β-xanthins were determined by measuring the absorbance at 540 and 475 nm, respectively, using a spectrophotometer (Hitachi, U-1800, Tokyo, Japan). The results of β-cyanins and β-xanthins were expressed by calculating, as nanograms, betanin and indicaxanthin equivalent per g of fresh weight for β-cyanins and β-xanthins, respectively.

### 2.9. Estimation of β-Carotene

We used our described method for the estimation of β-carotene [168,169]. In a mortar and pestle, 500 mg of fresh leaves was added with 10 mL acetone (80%), and ground precisely. The extract was centrifuged for 3–4 min at 10,000× *g*. After removing the supernatant in a volumetric flask, the final volume was marked up to 20 mL. A spectrophotometer (Hitachi, Tokyo, Japan) was used to take the absorbance at 510 nm and 480 nm. Data were expressed as milligrams of β--carotene per 100 g of fresh weight.

β-Carotene = 7.6 {(Abs. at 480 nm) − 1.49 (Abs. at 510 nm) × Final volume}/(1000 × fresh weight of leaf).

### 2.10. Determination of Ascorbic Acid

The fresh leaf samples were pre-incubated, and dehydroascorbic acid (DHA) was reduced to ascorbic acid (AsA) using Dithiothreitol (DTT). AsA reduced Fe^3+^ to Fe^2+^. 2, 2-dipyridyl forms complexes with reduced Fe^2+^ [170]. The optical density was taken using a spectrophotometer (Hitachi, Japan) at 525 nm to estimate ascorbic acid (AsA). We calculated ascorbic acid in milligrams per 100 g of fresh weight.

### 2.11. Samples Extraction and Determination of TP, TF, and RSC

The extraction was performed from the fresh and dried ground leaves (30 d) with a mortar and pestle for total polyphenols (TP) content, total flavonoids (TF) content, and RSC determination. In a tightly capped bottle, 0.25 g of leaves were added in 10 mL MeOH (90%), and placed in a shaking water bath (Tokyo, Japan). The extract was filtered after 1 h and stored for TP, TF, and RSC. Phenolic content was determined by the Folin–Ciocalteu reagent [170]. The optical density was taken at 760 nm using a spectrophotometer (HITACHI, Japan). TP was calculated as gallic acid equivalent μg GAE g^−1^ of FW using an equation (Y = 0.009X + 0.019) obtained from a standard gallic acid graph. The aluminum chloride colorimetric method was followed to estimate the total flavonoid content [170]. The optical density was measured at 415 nm using a spectrophotometer (HITACHI, Japan). A rutin standard graph (Y = 0.013X) was made to estimate TF as μg RE g^−1^ DW. The RSC was estimated by the diphenyl-picrylhydrazyl (DPPH) radical degradation method [171], and ABTS^+^ assay was carried out using the method of Sarker and Oba [172]. Percentage of inhibition of ABTS^+^ and DPPH equivalent to the control was applied to measure the RSC using following the equation:

RSC (%) = (AC − AS/AC) × 100(1)

where AC is the absorbance of the control (150 μL and 10 µL MeOH for RSC (ABTS), RSC (DPPH) instead of leaf extract), and AS is the absorbance of the samples. The results were calculated as μg Trolox equivalent g^−1^ DW.

### 2.12. Statistical Analysis

The replication mean was obtained by averaging the replication-wise row data. Analysis of variance (ANOVA) of the mean data was analyzed using Statistix 8 software [173,174,175,176]. Duncan Multiple Range Test at a 1% level of probability was followed to compare means data. The results were expressed as the mean ± SD. The correlation was analyzed using Statistix 8 software.

## 3. Results and Discussion

The variance analysis revealed that all the parameters significantly differed regarding the accessions, indicating a wide range of variations across the genotypes of two species of *Amaranthus* leafy vegetables. An extensive range of variability was also stated in red and green color amaranth [167].

### 3.1. Proximate Contents

The composition of the proximate (g 100 g^−1^ FW) of eight selected accessions of two underutilized species of *Amaranthus* leafy vegetables is shown in Table 1. The content of moisture of eight selected accessions of two underutilized *Amaranthus* leafy vegetable species varied from 82.85 to 88.52. AT2 displayed the highest moisture content (88.52) after *A. tricolor* genotype AT12 (86.43) and AT9 (86.38). Alternately, SA10 had the least moisture content (82.85), which was statistically similar to SA14, SA3, SA21, and AT5. Across eight accessions of two underutilized species, AT5, SA3, SA10, SA14, and SA21 showed low moisture content (18% dry matter). As lower moisture ensures a higher dry weight of leaves, these accessions could be used as superior dry biomass. The moisture content is directly related to the maturity of the leaves. The results obtained from underutilized species were corroborated by the reports of sweet potato leaves [177]. In comparison to leafy vegetables, the leaves of eight selected accessions of two underutilized species of *Amaranthus* leafy vegetables had good protein content that significantly varied regarding accessions (3.66 to 6.52). The highest protein content was recorded in SA14 (6.52). On the contrary, SA21 displayed the least protein content (3.66). Compared to leafy vegetables, higher protein content was obtained from AT2, AT5, AT9, AT12, SA3, and SA10. Vegetable amaranth is the primary source of protein for vegetarians and poor people in developing countries. The protein content of the accessions of two underutilized species was much more prominent as compared to *A tricolor* (1.26%) [20].

In this investigation, the selected accessions of two underutilized species displayed lesser fat, owing to leafy vegetables, and they might be utilized as foods free from cholesterol. AT12 confirmed the highest fat (0.35), although SA21 had the least fat (0.16) that had a statistical similarity with SA14. The results of sweet potato [177] were corroborated with our current results. They stated that fat covering the body’s organs influences cell function and perpetuation body temperature. Vegetable fats are the primary sources of essential fatty acids, such as omega-6 and omega-3. Fats play a significant role in the absorption, digestion, and transport of vitamins E, D, A, and K, which are soluble in fats. The selected accessions of two underutilized species confirmed good carbohydrate content with ample variations regarding accessions (0.38 to 9.56). SA21 confirmed the highest carbohydrates (9.56), though AT2 had the least carbohydrates (0.38). The accessions of two underutilized species were principally diverse for energy (28.95 to 58.47). The accession AT5, SA3, and SA14 demonstrated the highest energy (53.80, 53.81, and 53.82, respectively). On the other hand, AT2 displayed the least energy (25.11). AT5 and SA14 exhibited the highest ash (5.62 and 5.56, respectively), whereas AT9 showed the least ash (2.91). Fiber was largely diverse among accessions (6.66 to 10.24). AT9 confirmed the highest fiber (10.24) after AT5 and SA21. Inversely, SA14 had the least fiber (6.66). Fiber had a noteworthy involvement in the cure of constipation, the augmentation of digestibility, and palatability [19]. Our results displayed that those leaves of selected accessions of two underutilized species have copious protein, moisture, carbohydrates, and digestible fiber. The highest fat and fiber were obtained from *A. tricolor* accessions. Similarly, the highest protein and carbohydrates were obtained from *A. lividus* accessions. The highest dry matter, energy, and ash were obtained from both *A. tricolor* and *A. lividus* accessions. The moisture and protein contents received from accessions were superior to the moisture and protein contents of the green, red, stem, and weedy amaranth and *A. blitum* [178,179,180,181,182]. The carbohydrates of advanced line AT7 were greater than red, green amaranth, *A. spinosus*, and *A. blitum* [178,179,180,182], although the carbohydrates of AT7 were corroborative with stem amaranth [181]. The fiber of AT3 and AT7 were superior to red, stem, and green amaranth and *A. blitum* [178,180,181,182], although corroborated by weedy amaranth [179].

### 3.2. Mineral Content

Macroelements (mg g^−1^ FW) and microelements (µg g^−1^ FW) of eight selected accessions of two underutilized species of *Amaranthus* leafy vegetables are shown in Table 2. The selected accessions of two underutilized species demonstrated good K content. AT12 had the highest K content (6.72) after AT2, AT5, and AT9. On the contrary, the highest K content was recorded in SA14 and SA21 (3.77 and 3.72, respectively). SA3 showed the highest Ca content (3.45) after AT2, AT9, and AT12. Inversely, SA21 displayed the least Ca (1.56). The accessions of two underutilized species demonstrated good Mg content with prominent variations regarding accessions (2.65 to 3.74). AT12 confirmed the highest Mg (3.62). Inversely, AT7 confirmed the lowest Mg (3.74) after AT2, AT5, and AT9. It exposed that sufficient K (6.72), Mg (3.74), and Ca (3.45) were noted in the selected accessions of two underutilized species (based on fresh weight). Several species of amaranth literature [183] stated sufficient, Mg, Ca, and K. Furthermore, they detected that Ca, Mg, and K of amaranth were much more protuberant than nightshade, black spider flower, kale, and spinach. Mg, Ca, and K obtained from the accessions of two underutilized species were superior to Mg, Ca, and K of literature [183]. *A. tricolor* had the highest K and Mg. In contrast, the *A. lividus* demonstrated the highest Ca. K content of advanced lines was more than K of green amaranth [180], though K obtained from these advanced lines was inferior to K of weedy amaranth [179]. Ca content observed in the study was corroborative to the results of green and weedy amaranth [179,180]. Mg noticed in these accessions was greater than our previous green and weedy amaranth [179,180]. The protuberant differences were observed in Fe content regarding accessions (10.72 to 18.34). AT12 and SA10 displayed the highest Fe (18.34 and 18.24, respectively), though the least Fe was displayed in SA21 and AT5 (10.72 and 10.76, respectively). Our study revealed that preponderant variations were noticed.

In Mn content of the selected accessions of two underutilized species (3.12 to 15.12), AT12 had the highest Mn (15.12), although the least Mn was recorded in SA3 (3.12). The Cu had an extensive array of variations in the accessions of two underutilized species (1.04 to 2.98). SA3 confirmed the highest Cu (2.98), although AT9 and AT5 exerted the least Cu (1.04 and 1.05, respectively). Zn of the accessions of two underutilized species differed significantly and markedly (6.02 to 17.12). AT12 confirmed the highest Cu (17.12), whereas SA14 exerted the least Cu (6.02). The Fe and Zn content of the accessions of two underutilized species was superior to the leaves of cassava [184] and beach pea [185]. We documented sufficient Fe (18.34), Mn (15.12), Zn (17.12), and noteworthy Cu (2.98) (based on fresh weight) in the accessions of two underutilized species. Similarly, different amaranths in literature [183] observed satisfactory Fe, Mn, Cu, and Zn. They also stated that Fe, Zn, Mn, and Cu in the leaves of amaranth were superior to spinach, spider flower, black nightshade, and kale. Mn, Fe, Zn, and Cu in the study were superior to Mn, Fe, Zn, and Cu in the literature [183]. *A. tricolor* accessions confirmed the highest Mn and Zn. Similarly, *A. lividus* accessions confirmed the highest Cu. *A. tricolor* and *A. lividus* accessions confirmed the highest Fe. In the study, Fe and Mn of all advanced lines were superior to green amaranth [180], although Fe and Mn of AT15 were superior to weedy amaranth [179]. Cu observed in the study was superior to green and weedy morph amaranth [179,180] and *A. spinosus* amaranth [179]. AT15 displayed much greater Zn than green and weedy amaranth [179,180].

### 3.3. Antioxidant Phytopigment Content

Chlorophylls (μg g^−1^ FW), and betalains (ng g^−1^ FW) of the selected accessions of two underutilized species are shown in Table 3. Prominent variations in chlorophyll *a* were stated among accessions of two underutilized species (134.15 to 636.56). AT5 demonstrated the highest chlorophyll *a* (636.56) after AT2 and AT12. Inversely, the least chlorophyll *a* (134.15) was observed in AT3.

Selected accessions of two underutilized species demonstrated wide variations in chlorophyll *b* content (82.71 to 285.32). SA14 confirmed the highest chlorophyll *b* (285.32) after AT5. In contrast, SA10 confirmed the least chlorophyll *b* (82.71). Noteworthy and outstanding variations in chlorophyll *ab* were confirmed in the accessions of two underutilized species (216.86 to 913.20). Across the two underutilized species, AT5 exhibited the highest chlorophyll *ab* (913.20) after AT2, although SA10 confirmed the least chlorophyll *ab* (216.86). Notably, chlorophyll *a*, *ab*, and *b* (636.56, 913.20, and 285.32) in the accessions of two underutilized species, were superior to red and green amaranth [186]. *A. tricolor* accessions confirmed the highest chlorophyll *a* and *ab*. Similarly, *A. lividus* accessions confirmed the highest chlorophyll *b*. Chlorophylls *a*, *ab*, and *b* of the study were superior to red, green, stem, and weedy amaranth and *A. blitum* [178,179,180,181,182].

The accessions of two underutilized species established good β-cyanins content with significant variability among accessions (192.44 to 542.75). AT9 confirmed the highest β-cyanins (542.75) after AT2. Inversely, SA10 showed the least β-cyanins (192.44 ng g^−1^). The selected accessions of two underutilized species established good β-xanthins with significant variability regarding accessions (196.55 to 596.73). AT9 confirmed the highest β-xanthins (596.73) after SA3. Inversely, SA10 showed the least β-xanthins (196.55). The accessions of two underutilized species established good content of betalains with protruding variability among accessions (388.99 to 1139.48). The betalains were the highest in AT9 (1139.48) after SA3. Inversely, the least betalains were stated in SA10 (388.99). Moreover, carotenoids and betalains showed major variability in the accessions of two underutilized species (45.44 to 123.28). AT12 and SA21 confirmed the highest carotenoids (123.28 and 122.94). Inversely, SA3 confirmed the least carotenoids (45.44). Notably, chlorophyll *a*, *ab*, and *b* (636.56, 913.20, 285.32); β-cyanins (542.75); β-xanthins (596.73); betalains (1139.48); and carotenoids (123.28), in the accessions of two underutilized species, were corroborated to green and red amaranth [186]. β-cyanins, β-xanthins, and betalains pigments were the highest in *A. tricolor* accessions. Inversely, the highest carotenoids were obtained from both *A. tricolor* and *A. lividus*. Betalains, β-cyanins, and β-xanthins in the study were superior to red, green, stem amaranth, and *A. blitum* [178,180,181,182]. Carotenoids in the study were superior to green amaranth [180], and verified to weedy amaranth [179], although the carotenoids of this study were inferior to red and stem amaranth and *A. blitum* [178,181,182].

### 3.4. Phytochemical Contents and Scavenging Activity

TP, β-carotene, TF, ascorbic acid, and RSC of the selected accessions of two underutilized species are shown in Table 4. Pronounced variability was recorded in the β-carotene content of the selected accessions of two underutilized species (27.67 in AT5 to 68.82 in AT12).

AT2 and SA10 confirmed the high β-carotene. The accessions of two underutilized species demonstrated prominent variations in ascorbic acid (18.54 to 192.75). AT2 confirmed the highest ascorbic acid (192.75), and the least in AT9 (18.54). Noteworthy variations were noted in TP of the accessions of two underutilized species (15.66 to 32.88). AT5 established the highest TP of 32.88 after AT2 and AT9. Conversely, SA10 showed the least TP (15.66). The accessions of two underutilized species demonstrated high TF with substantial variability among accessions (140.71 to 176.88). AT12 showed the highest TF (176.88) after AT5, whereas SA14 had the least TF (140.71). The accessions of two underutilized species confirmed high RSC (DPPH) and RSC (ABTS^+^). AT2 showed the highest RSC (DPPH and ABTS^+^) (36.27, 68.87) after AT5 (35.08, 64.55) and AT9 (33.84, 64.56). On the other hand, the least RSC (DPPH) and RSC (ABTS^+^) were recorded in SA3 (22.75, 40.86) after SA10 (24.45, 42.44). A parallel tendency of RSC (DPPH) and RSC (ABTS^+^) methods authenticated the measurement of two different methods of AP. The accessions of two underutilized species exhibited outstanding ascorbic acid and β-carotene (192.75 and 68.82). TP (32.88), TF (176.88), RSC (DPPH) (36.27), and RSC (ABTS^+^) (68.87) found in this study were superior to red and green amaranth [187]. The β-carotene of these lines was confirmative to weedy amaranth [179]. The ascorbic acid of AT11 was superior to green, weedy, stem, amaranth, and *A. blitum* [179,181,182], and corroborative to red morph amaranth [178]. TP content was greater than green and weedy amaranth [179,180]. TF, RSC (DPPH), and RSC (ABTS^+^) documented in the accessions of two underutilized species were greater than the red, green, stem, and weedy amaranth and *A. blitum* morph [178,179,180,181,182]. The accession AT2 had the highest vitamin C and RSC (DPPH) and RSC (ABTS^+^); AT5 had the highest TP content; and AT12 had the highest TF content. *A. tricolor* accessions had high phytochemicals across the two species, such as phytopigments, vitamins, phenolics, flavonoids, and antioxidants, including considerable nutrients and protein. *A. tricolor* accessions can be used as high-yielding cultivars comprising ample antioxidants. The accessions confirmed an immense foundation of phytochemicals, and nutritional values and AP presented enormous prospects for feeding the mineral-, vitamin-, and antioxidant-deficient community.

### 3.5. The Correlation Studies

The correlation of β-carotene, pigments, TP, ascorbic acid, TF, and RSC (ABTS^+^) and RSC (DPPH) of accessions of two underutilized species are shown in Table 5, representing exciting results. All pigments confirmed positive and significant correlations with TP, TF, and RSC (ABTS^+^) and RSC (DPPH), indicating that the increase in TF, TP, and RSC (DPPH) and RSC (ABTS^+^) were straightly linked to the augmentation of chlorophylls, β-cyanins, carotenoids, betalains, and β-xanthins or vice versa. Its destined pigments had good RSC. Similarly, β-carotene had a significant positive relationship with TP, ascorbic acid, TF, and RSC (ABTS^+^) and RSC (DPPH), although it exhibited significant negative associations among all pigments. Ascorbic acid had a significant positive relationship with TP, TF, and RSC (ABTS^+^) and RSC (DPPH), although it displayed insignificant negative associations among pigments. In amaranth, Sarker, and Oba [142] also observed a similar trend. A significant positive association was displayed among TP, TF, and RSC (ABTS^+^) and RSC (DPPH), which is corroborative to the results of amaranth and salt stressed-purslane [187,188,189,190]. The validation of the antioxidant capacity of the selected advanced lines of vegetable amaranth by two different methods of antioxidant capacity measurements was confirmed with the significant positive associations between RSC (ABTS^+^) and RSC (DPPH).

Phytochemicals and pigments including β-carotene, TP, ascorbic acid, and TF had intense AP, as these showed significant associations with RSC (DPPH) and RSC (ABTS^+^).

## 4. Conclusions

The selected accessions of two underutilized species demonstrated leafy vegetables as abundant sources of K, Fe, Mn, Ca, dry matter, Cu, Zn, protein, Mg, dietary fiber, and carbohydrates. It is an excellent origin of antioxidant pigments and phytochemicals, including TP, β-carotene, TF, ascorbic acid, and antioxidants. The accessions of *A. tricolor* had abundant phytochemicals and RSC, including considerable proximate, pigments, and nutraceuticals compared to the accessions of *A. lividus*. The interrelationship exposed that phytochemicals and pigments of leafy vegetable amaranth accessions confirmed good RSC of 2,2-azino-bis(3-ethylbenzothiazoline-6-sulfonic acid) and 2,2-Diphenyl-1-picrylhydrazyl equivalent to Trolox. Although *A. tricolor* and *A. lividus* are underutilized, these are promising leafy vegetables. Enormous bioactive phytochemicals and antioxidants of *A. tricolor* and *A. lividus* enable growing them as preferable cultivars, and they can be used in the daily diet as fresh salad, boiled, leafy vegetables, and other culinary dishes. Based on their nutritional status, they are comparable to spinach, and can be grown throughout the year, including a gap period of leafy vegetables in summer. They are a potential source of nutritional value, antioxidant phytopigments, β-carotene, ascorbic acid, phenolics, flavonoids, and antioxidants in our daily diet to accomplish nutritional and antioxidant sufficiency.

## Figures and Tables

**Table 1:** Proximate compositions (g 100 g^−1^ fresh weight) of two underutilized species of *Amaranthus* leafy vegetables.

Accessions	Water (%)	Crude Protein	Crude Fat	Carbohydrate	Ash	Energy (kcal)	Fiber
*A. tricolor*							
AT2	88.52 ± 1.86 a	5.35 ± 0.04 b	0.27 ± 0.04 b	0.38 ± 0.05 h	5.48 ± 0.02 ab	25.11 ± 0.32 d	7.19 ± 0.15 d
AT5	81.92 ± 1.44 c	5.33 ± 0.05 b	0.33 ± 0.03 a	6.50 ± 0.06 d	5.62 ± 0.02 a	53.80 ± 0.31 a	9.22 ± 0.17 b
AT9	86.38 ± 1.35 b	5.37 ± 0.06 b	0.29 ± 0.03 ab	5.05 ± 0.07 f	2.91 ± 0.02 d	43.81 ± 0.38 c	10.24 ± 0.13 a
AT12	86.43 ± 1.35 b	5.32 ± 0.04 b	0.35 ± 0.04 a	4.07 ± 0.06 g	3.83 ± 0.03 c	40.71 ± 0.26 c	8.34 ± 0.12 c
*A. lividus*							
SA3	81.86 ± 1.56 c	5.35 ± 0.05 b	0.27 ± 0.04 b	7.00 ± 0.05 c	5.52 ± 0.04 ab	53.81 ± 0.36 a	8.56 ± 0.17 c
SA10	81.85 ± 1.38 c	5.34 ± 0.03 b	0.23 ± 0.03 bc	7.46 ± 0.06 b	5.12 ± 0.03 b	52.55± 0.42 ab	7.76 ± 0.15 cd
SA14	81.87 ± 1.51 c	6.52 ± 0.04 a	0.18 ± 0.03 c	5.87 ± 0.06 e	5.56 ± 0.04 a	53.82 ± 0.35 a	6.66 ± 0.15 e
SA21	81.91 ± 1.62 c	3.66 ± 0.04 c	0.16 ± 0.03 c	9.56 ± 0.05 a	4.71 ± 0.02 d	51.60 ± 0.36 b	9.18 ± 0.12 b
Significance	**	**	**	**	**	**	**
CV%	2.36	1.35	0.24	0.66	0.55	0.75	0.38

CV, coefficient of variation; in a column, different letters in mean values are significantly differed by Duncan Multiple Range Test (**, *p* < 0.01); *n* = 3.

**Table 2:** Macroelements (mg g^−1^ FW) and microelements (µg g^−1^ FW) of two underutilized species of *Amaranthus* leafy vegetables.

Genotypes	Macroelements			Microelements			
	K	Ca	Mg	Fe	Mn	Cu	Zn
*A. tricolor*							
AT2	4.96 ± 0.11 b	2.32 ± 0.15 b	3.38 ± 0.17 b	11.86 ± 0.26 c	6.86 ± 0.13 c	1.32 ± 0.03 d	6.85 ± 0.13 e
AT5	4.97 ± 0.10 b	1.93 ± 0.16 c	3.39 ± 0.13 b	10.76 ± 0.23 e	7.92 ± 0.17 b	1.05 ± 0.02 e	7.08 ± 0.15 d
AT9	4.95 ± 0.14 b	2.36 ± 0.15 b	3.42 ± 0.18 b	15.05 ± 0.28 b	6.79 ± 0.14 c	1.04 ± 0.03 e	7.06 ± 0.16 d
AT12	6.72 ± 0.11 a	2.35 ± 0.11 b	3.74 ± 0.12 a	18.34 ± 0.25 a	15.12 ± 0.18 a	2.12 ± 0.03 c	17.12 ± 0.14 a
*A. lividus*							
SA3	4.22 ± 0.14 c	3.45 ± 0.17 a	3.15 ± 0.19 c	11.08 ± 0.24 d	3.12 ± 0.12 g	2.98 ± 0.04 a	7.05 ± 0.13 d
SA10	4.15 ± 0.16 c	1.88 ± 0.15 c	3.35 ± 0.13 b	18.24 ± 0.21 a	3.76 ± 0.14 f	2.32 ± 0.03 b	8.72 ± 0.18 b
SA14	3.77 ± 0.14 d	1.91 ± 0.16 c	2.98 ± 0.17 c	11.02 ± 0.23 d	4.77 ± 0.14 e	1.34 ± 0.03 d	6.02 ± 0.12 f
SA21	3.72 ± 0.16 d	1.56 ± 0.16 cd	2.65 ± 0.14 d	10.72 ± 0.17 e	5.95 ± 0.14 d	2.08 ± 0.05 c	7.86 ± 0.15 c
Significance	**	**	**	**	**	**	**
CV%	0.12	0.35	0.25	0.21	0.32	0.13	0.26

CV, coefficient of variation; *n* = 3; in a column, different letters in mean values are significantly differed by Duncan Multiple Range Test (**, *p* < 0.01).

**Table 3:** The performance of the mean antioxidant pigments (chlorophylls (μg g^−1^ FW); β-Cyanins, β-xanthins, and betalains (ng g^−1^ FW); carotenoids (mg 100 g^−1^ FW)) of two underutilized species of *Amaranthus* leafy vegetables.

Genotypes	Chlorophyll *a*	Chlorophyll *b*	Chlorophyll *ab*	β-Cyanins	β-Xanthins	Betalains	Carotenoids
*A. tricolor*							
AT2	519.55 ± 2.11 b	264.52 ± 1.68 c	784.07 ± 1.25 b	536.32 ± 1.61 b	528.42 ± 1.71 c	1064.74 ± 1.54 c	72.68 ± 0.37 d
AT5	636.56 ± 2.14 a	276.64 ± 1.77 b	913.20 ± 1.21 a	417.34 ± 1.63 e	434.65 ± 1.63 e	851.99 ± 1.64 e	82.65 ± 0.35 c
AT9	308.72 ± 2.02 e	212.72 ± 1.78 f	521.44 ± 1.14 f	542.75 ± 1.64 a	596.73 ± 1.95 a	1139.48 ± 1.44 a	95.35 ± 0.37 b
AT12	518.59 ± 2.16 b	228.24 ± 1.78 e	746.83 ± 1.18 d	489.58 ± 1.62 d	498.66 ± 1.88 d	988.24 ± 1.47 d	123.28 ± 0.42 a
*A. lividus*							
SA3	432.35 ± 2.17 d	242.16 ± 1.62 d	674.51 ± 1.25 e	524.56 ± 1.72 c	558.35 ± 1.52 b	1082.91 ± 1.65 b	45.44 ± 0.41 f
SA10	134.15 ± 2.32 g	82.71 ± 1.61 h	216.86 ± 1.12 h	192.44 ± 1.58 h	196.55 ± 1.42 h	388.99 ± 1.42 h	82.57 ± 0.42 c
SA14	478.45 ± 2.17 c	285. 32 ± 1.73 a	763.77 ± 1.23 c	292.62 ± 1.72 f	292.25 ± 1.37 f	554.87 ± 1.57 f	58.74 ± 0.42 e
SA21	256.26 ± 2.22 f	93.33 ± 1.69 g	349.53 ± 1.31 g	243.24 ± 1.6 g	265.86 ± 1.68 g	509.10 ± 1.72 g	122.94 ± 0.45 a
Significance	**	**	**	**	**	**	**
CV%	3.55	1.28	1.46	2.16	2.35	1.23	2.33

CV, coefficient of variation; In a column, different letters in mean values are significantly differed by Duncan Multiple Range Test (**, *p* < 0.01); *n* = 3.

**Table 4:** The performance of TP (µg GAE g^−1^ FW), vitamin C (mg 100 g^−1^ FW), β-Carotene (mg 100 g^−1^ FW), RSC (DPPH) (µg g^−1^ TEAC DW), TF (µg RE g^−1^ DW), and RSC (ABTS^+^) (µg TEAC g^−1^ DW) of two underutilized species of *Amaranthus* leafy vegetables.

Genotypes	β-Carotene	Vitamin C	TP	TF	RSC (DPPH)	RSC (ABTS^+^)
*A. tricolor*						
AT2	62.58 ± 0.65 b	192.75 ± 1.48 a	29.48 ± 0.42 b	158.84 ± 1.26 c	36.27 ± 0.11 a	68.87 ± 0.34 a
AT5	27.67 ± 0.56 g	98.56 ± 1.52 c	32.88 ± 0.38 a	172.55 ± 1.29 b	35.08 ± 0.12 b	64.55 ± 0.31 b
AT9	48.55 ± 0.62 d	18.54 ± 1.49 h	29.45 ± 0.37 b	151.88 ± 1.36 d	33. 84 ± 0.09 c	64.56 ± 0.39 b
AT12	68.82 ± 0.69 a	72.98 ± 1.46 e	22.43 ± 0.45 e	176.88 ± 1.28 a	33.85 ± 0.11 c	60.88 ± 0.38 c
*A. lividus*						
SA3	32.74 ± 1.68 f	58.74 ± 1.48 g	18.62 ± 0.42 f	151.75 ± 1.24 d	22.75 ± 0.11 g	40.86 ± 0.37 g
SA10	61.83 ± 1.66 b	65.84 ± 1.52 f	15.66 ± 0.42 g	148.56 ± 1.25 cd	24.45 ± 0.15 f	42.44 ± 0.41 f
SA14	39.35 ± 1.58 e	175.87 ± 1.45 b	26.24 ± 0.36 c	140.71 ± 1.23 e	26.23 ± 0.14 e	48.95 ± 0.46 e
SA21	54.74 ± 1.56 c	87.76 ± 1.58 d	25.45 ± 0.42 d	157.93 ± 1.28 c	28.86 ± 0.18 d	55.76 ± 0.48 d
Significance	**	**	**	**	**	**
CV%	1.53	2.26	3.72	1.32	1.63	0.87

CV, coefficient of variation; TF = total flavonoid content, RSC = radical scavenging capacity, TP = total polyphenol content, *n* = 3; in a column, mean values with different letters are differed significantly by Duncan Multiple Range Test (**, *p* < 0.01).

**Table 5:** The coefficient of correlation of antioxidant pigments, TPC (µg GAE g^−1^ FW), β-Carotene (mg 100 g^−1^ FW), vitamin C (mg 100 g^−1^ FW), RSC (DPPH) (µg g^−1^ TEAC DW), TFC (µg RE g^−1^ DW), and RSC (ABTS^+^) (µg TEAC g^−1^ DW), chlorophylls (μg g^−1^ FW); β-Cyanins, β-xanthins, and betalains (ng g^−1^ FW); carotenoids (mg 100 g^−1^ FW) of two underutilized species of *Amaranthus* leafy vegetables.

Traits	Chl *b*	Chl *ab*	β-Cyanins	β-Xanthins	Betalains	β-Carotene	Vitamin C	TP	TF	RSC (DPPH)	RSC (ABTS^+^)
Chlorophyll *a*	0.86 **	0.92 **	0.89 **	0.85 **	0.82 **	−0.73 *	−0.015	0.77 *	0.75 *	0.86 **	0.86 **
Chlorophyll *b*		0.85 **	0.77 **	0.85 **	0.86 **	−0.67	−0.018	0.72 *	0.74 *	0.84 **	0.84 **
Chlorophyll *ab*			0.72 *	0.76 *	0.83 **	−0.76*	−0.014	0.75 *	0.82 **	0.73 *	0.85 **
β-cyanins				0.87 **	0.96 **	−0.75 *	−0.116	0.76 *	0.81 **	0.88 **	0.87 **
β-xanthins					0.95 **	−0.86 **	−0.123	0.74 *	0.72*	0.76 *	0.83 **
Betalains						−0.88 **	−0.129	0.86 **	0.77*	0.84 **	0.88 **
β-Carotene							0.73*	0.82 **	0.88 **	0.86 **	0.85 **
Vitamin C								0.74 *	0.86 **	0.85 **	0.86 **
TP									0.84 **	0.82 **	0.92 **
TF										0.86 **	0.86 **
RSC (DPPH)											0.95 **

Chl *b*, chlorophyll *b;* TP, total polyphenol content; TF, total flavonoid content; RSC, radical scavenging capacity; total antioxidant capacity; Chl *ab*, chlorophyll *b*; *, **, significant at 5% and 1% level.

## Data Availability

Data recorded in the current study are available in all Tables of the manuscript.
